# Impact of excessive phone usage on hand functions and incidence of hand disorders

**DOI:** 10.1097/MS9.0000000000003143

**Published:** 2025-03-07

**Authors:** Maryam Athar, Javeria Hashmi, Muhammad Meeran Saleem, Javeria Azam, Shahood Ahmed Umar, Mohammed Mahmmoud Fadelallah Eljack

**Affiliations:** a DOW Medical College, DOW University of Health Sciences, Karachi, Pakistan; b DOW Medical College, Jinnah Sindh Medical University, Karachi, Pakistan; c DOW Medical College, Ziauddin Medical College, Karachi, Pakistan; d Community Department, University of Bakht Alruda, Ad Duwaym, Sudan

**Keywords:** carpal tunnel syndrome (CTS), De Quervain’s tenosynovitis (DQT), hand disorders, smartphone

## Abstract

Smartphone usage has become ubiquitous, with over half the global population owning these devices. However, excessive smartphone use has been linked to various hand-related disorders. This short communication explores the impact of prolonged phone use on hand function and its association with musculoskeletal disorders like carpal tunnel syndrome (CTS) and De Quervain’s tenosynovitis (DQT). CTS, resulting from median nerve compression, is prevalent among smartphone users who engage in repetitive wrist and finger movements. Likewise, DQT, caused by inflammation of thumb tendons, is increasingly observed in individuals who frequently use their thumbs for texting and gaming. Studies indicate that prolonged daily smartphone usage correlates with diminished grip strength, increased wrist pain, and a heightened risk of developing CTS and DQT. Young adults, particularly university students, are disproportionately affected, with over 50% of heavy smartphone users reporting hand pain or dysfunction. This communication highlights the need for ergonomic interventions, awareness of proper smartphone use, and targeted prevention strategies to mitigate these risks. Addressing factors like poor posture, prolonged device use, and thumb-dominated activities can help prevent long-term hand disorders. By recognizing these health risks and taking proactive measures, individuals can reduce the incidence of hand dysfunction related to excessive phone use and improve their overall hand health.

Over the past 10 years, smartphone ownership has grown significantly, now exceeding around half of the world’s population^[1]^. A study indicates that 79% of people aged 18–44 spend most of their time on smartphones^[2]^. Amid the advantages of smartphone usage lie significant drawbacks: excessive smartphone use can result in elevated levels of anxiety and depression^[3]^, alongside diminished conversation quality and connection with others^[4]^. Students also experience headaches and concentration interruptions from constant messages and calls, affecting their coursework completion^[5]^. The widespread use of smartphones has become a common practice, exposing users to various health concerns. One such example is decreased hand and pinch grip strength, which affects overall hand performance as well^[6]^.

**HIGHLIGHTS**

- Recent studies have shown an increasing usage of smartphones around the world and adverse effects caused by their excessive usage.
- Research has demonstrated a direct correlation between smartphone usage and the severity of wrist discomfort and skeletomuscular disorders affecting the hands and wrists.
- Excessive smartphone usage can give birth to disorders such as carpal tunnel syndrome, tendinosis, and De Quervain’s tenosynovitis.
- Preventive measures, such as reducing the overall usage time, frequent short breaks between usage, maintaining correct posture might be effective in preventing such disorders.

Over usage of smartphones has been linked with multiple skeletomuscular pathologies frequently involving the hand and wrist^[7]^. Musculoskeletal disorders are induced by sudden strain or extended exposure to physical variables such as extended, vigorous, and repetitive usage of mobile devices^[8]^. One study shows that 27.2% of university students spend more than 8 hours per day using their smartphones. This finding may prompt concerns regarding the risk of musculoskeletal disorders among university students^[9]^. Another cross-sectional survey conducted in the universities of Islamabad and Rawalpindi, including students aged between 18 and 25, revealed that the intensity of wrist pain and impairment rises directly with the extent of mobile phone usage^[10]^. The findings of this study suggested that excessive mobile phone users suffer from higher levels of wrist pain and discomfort. Both high and low-level users show reduced strength in their dominant hands, with a significant association with hand disorders like carpal tunnel syndrome (CTS) and De Quervain’s tenosynovitis (DQT). This underscores the direct link between heavy smartphone use and these specific hand conditions^[11]^.

CTS is the most common peripheral nerve compression disorder, impacting nearly 3.8% of the general population^[12]^. The compression of the median nerve in the carpal tunnel compromises the blood-nerve barrier, resulting in edema, inflammation, and fibrosis of the adjacent connective tissues. The subsequent stage of the illness involves disruption of the myelin sheath of the nerve followed by damage to the axon. Patients typically exhibit numbness and pain in the palmar surfaces of the first, second, third, and radial half of the fourth finger, experience nocturnal pain, and observe a gradual progression of their symptoms^[13]^. CTS renders the ability to use the hand effectively, making it difficult for people to complete tasks and hindering their everyday lives and employment. Physiological evidence shows that elevated pressure within the carpal tunnel disrupts the role of the median nerve at that site, leading to diminished grip strength and reduced functionality of the affected hand^[14]^. Risk factors for CTS include extended periods of severe wrist flexion or extension, frequent use of the flexor muscles, and exposure to vibrations; all of which are commonly associated with smartphone use^[15]^. Multiple studies have demonstrated the connection between excessive smartphone use and CTS, identifying it as a common industrial illness among smartphone users^[16,17]^. A cross-sectional study conducted at Fırat University Faculty of Medicine revealed that the risk of CTS increases by 1.292 times for each additional hour of daily smartphone use. Consequently, smartphone addiction can be viewed as a significant risk factor for CTS^[17]^. Additionally, the incidence of CTS strongly correlates with smartphone use for 2 hours or longer daily^[16]^. A possible explanation may be that sustained smartphone use with awkward wrist positions, leads to excessive strain and elevated pressure within the carpal tunnel, leading to compression, deformation, rotation, and displacement of the median nerve and flexor pollicis longus tendon, resulting in hand musculoskeletal symptoms^[18]^.

DQT refers to a condition that impacts the tendons responsible for thumb movement, particularly impacting the abductor pollicis longus and extensor pollicis brevis tendons^[19]^. As the primary functionality of a smartphone is typically controlled by the user’s thumb(s), repeated exertion of thumbs and fingers can result in DQT^[20]^. A cross-sectional study evaluating smartphone usage patterns and their link to DQT among college students revealed that more than half (52%) of the students suffered from DQT. The study found that increased smartphone usage, including time spent on phone games, social media, and recreational activities, was associated with a higher risk of developing DQT among college students^[21]^. Another analytical cross-sectional study involving individuals aged 18–25 identified a correlation between daily smartphone usage duration and pain associated with excessive use. The study suggests that the level of smartphone use is directly proportional to the prevalence of DQT symptoms among young adults^[22]^. There also seems to be a direct correlation between the frequency of text messaging^[23]^ and mobile gaming^[24]^ and the incidence of DQT. This condition is notably more frequent among women, especially those aged 30–50 and during the postpartum period, particularly 4–6 weeks after childbirth^[25]^.

Apart from the aforementioned disorders, overuse of smartphones can lead to finger deformities and limited hand function, such as digital extensor tenosynovitis^[26]^, as well as causing weaker handgrip and pinch-grip strength in young people^[6]^. A retrospective analysis found that excessive use of handheld devices led to tendinosis of the Extensor Pollicis Longus and Myofascial Pain Syndrome. All subjects reported pain in the thumb and forearm, accompanied by burning sensation, numbness, and tingling in the thenar region of the hand, as well as hand and wrist rigidity^[27]^.

Smartphone usage patterns vary significantly among different age groups, potentially leading to problems. Older adults tend to exhibit dependent use based on call frequency, while younger users are more influenced by the time spent on phones and social media. This disparity highlights the need for targeted prevention efforts, especially for younger populations who are more prone to smartphone addiction^[28]^. Conversely, young adolescents (15–16 years) show a higher prevalence of smartphone dependency relative to young adults (19 years and older)^[29]^. Understanding these patterns is crucial for developing effective strategies to address problematic phone use across different age groups. Apart from age, other factors influencing the adverse effects of excessive mobile phone usage are posture while using the phone (standing or sitting), type of mobile phone task (texting, scrolling, etc.), and gender; standing while texting increases trapezius muscle activity more than sitting, with females showing higher muscle activity, increased thumb abduction, greater thumb movement speeds, and lesser pauses, while frequent swiping, scrolling, and tapping also strain the hand and wrist^[30]^. Making ergonomic design, mindful smartphone use, and gender-specific prevention efforts are crucial for mitigating these risks. Another study found that handheld devices that encourage the primary use of the thumb or a single finger for texting or gaming are linked to a higher incidence of musculoskeletal disorders. Therefore, users are advised to choose devices designed for typing or operating with all digits. Other preventive measures, such as reducing the overall usage time, tracking usage through apps, frequent short breaks between usage, maintaining correct posture, increasing in-person interactions, and using speech-to-text software might also be effective. Furthermore, the study found that a structured rehabilitation protocol was a beneficial treatment for such musculoskeletal disorders^[31]^.

One study suggested that factors like low self-esteem, impulsivity, and lack of family connections and friendships all contribute to excessive smartphone use^[32]^. Thus, intervention programs such as family communication programs would benefit both parents and children. An online randomized controlled trial revealed that mindfulness exercises, self-monitoring, and mood tracking can effectively reduce smartphone distraction^[33]^.Together, these measures create a multifaceted approach that can effectively mitigate the risks associated with excessive phone use.

In conclusion, the rapid rise in smartphone usage, particularly among younger populations, has brought about significant health concerns, especially regarding hand functions and the incidence of hand disorders. Excessive smartphone use is linked to diminished hand and pinch-grip strengths and several musculoskeletal disorders such as CTS and DQT. It is, thus, essential to undertake preventative measures as well as look towards developing more curative methods to counter this emerging problem.

## Data Availability

No new data was generated or analyzed in this paper.

## References

[1] Olson JA Sandra DA Colucci ÉS Smartphone addiction is increasing across the world: a meta-analysis of 24 countries. Comput Human Behav 2022;129:107138.

[2] Vate-U-Lan P. Text neck epidemic: a growing problem for smart phone users in Thailand. Int J Comp Internet Manag 2015;23:551–6.

[3] Hwang KH Yoo YS Cho OH. Smartphone overuse and upper extremity pain, anxiety, depression, and interpersonal relationships among college students. J Korea Contents Assoc 2012;12:365–75.

[4] Przybylski AK Weinstein N. Can you connect with me now? How the presence of mobile communication technology influences face-to-face conversation quality. J Soc Personal Relationships 2013;30:237–46.

[5] Abu-Shanab E Haddad E. The influence of smart phones on human health and behavior: Jordanians’ perceptions. Int J Comput Netw Appl 2015;2:52–56.

[6] Osailan A. The relationship between smartphone usage duration (using smartphone’s ability to monitor screen time) with hand-grip and pinch-grip strength among young people: an observational study. BMC Musculoskelet Disord 2021;22:186. 33588812 10.1186/s12891-021-04054-6 PMC7885228

[7] Al-Dhafer B Alessa HA Albesher MA The association between smartphone addiction/overuse with hand and wrist musculoskeletal complaints, Saudi Arabia. Cureus 2023;15:e38095. 38094550 10.7759/cureus.48752 PMC10718507

[8] Sharan D Ajeesh PS. Risk factors and clinical features of text message injuries. Work 2012;41:1145–48. 22316873 10.3233/WOR-2012-0294-1145

[9] Alosaimi F Alyahya H Alshahwan H Smartphone addiction among university students in Riyadh, Saudi Arabia. Saudi Med J 2016;37:675. 27279515 10.15537/smj.2016.6.14430 PMC4931650

[10] Amjad F Farooq MN Batool R Frequency of wrist pain and its associated risk factors in students using mobile phones. Pak J Med Sci 2020;36:746. 32494267 10.12669/pjms.36.4.1797 PMC7260896

[11] Saito K Saito Y. Relationship between information and communication device usage and development of hand disorders. Inquiry 2021;58:469580211029607. 34229528 10.1177/00469580211029607 PMC8267032

[12] Atroshi I Gummesson C Johnsson R Prevalence of carpal tunnel syndrome in a general population. jamanetwork.com. JAMA 1999;282:153–8. 10411196 10.1001/jama.282.2.153

[13] Pritsch T Rosenblatt Y Carmel A. Carpal tunnel syndrome. Harefuah 2004;143:743–64. 15521353

[14] Carpal Tunnel Syndrome - Clinical Practice Guideline (CPG). American Academy of Orthopaedic Surgeons. Accessed July 6, 2024. https://www.aaos.org/quality/quality-programs/upper-extremity-programs/carpal-tunnel-syndrome/.

[15] Pelmear P Taylor W. Carpal tunnel syndrome and hand-arm vibration syndrome: a diagnostic enigma. Arch Neurol 1994;51:416–20. 8155019 10.1001/archneur.1994.00540160118015

[16] Al Shahrani ES Al Shehri NA. Association between smartphone use and carpal tunnel syndrome: a case-control study. J Family Med Prim Care 2021;10:2816. 34660411 10.4103/jfmpc.jfmpc_2458_20 PMC8483076

[17] Karaçorlu FN Balgetir F Pirinçci E The relationship between carpal tunnel syndrome, smartphone use, and addiction: a cross-sectional study. Turk J Phys Med Rehabil 2022;68:517. 36589353 10.5606/tftrd.2022.9365 PMC9791704

[18] Woo H White P Ng H Development of kinematic graphs of median nerve during active finger motion: implications of smartphone use. PloS one 2016;11:e0158455. 27367447 10.1371/journal.pone.0158455 PMC4930216

[19] Fakoya AO Tarzian M Sabater EL De Quervain’s disease: a discourse on etiology, diagnosis, and treatment. Cureus 2023;15:e38079. 37252462 10.7759/cureus.38079 PMC10208847

[20] Morgan SD Sivakumar BS An VG A review of De Quervain’s stenosing tenovaginitis in the context of smartphone use. J Hand Surg Asian Pac Vol 2020;25:133–36. 32312208 10.1142/S2424835520300029

[21] Nie X Huang L Hou J Smartphone usage behaviors and their association with De Quervain’s Tenosynovitis (DQT) among college students: a cross-sectional study in Guangxi, China. BMC Public Health 2023;23:2257. 37974168 10.1186/s12889-023-16808-z PMC10652590

[22] Benites-Zapata VA Jiménez-Torres VE Ayala-Roldán MP. Problematic smartphone use is associated with de Quervain’s tenosynovitis symptomatology among young adults. Musculoskelet Sci Pract 2021;53:102356. 33667881 10.1016/j.msksp.2021.102356

[23] Ashurst J Turco D Lieb BE Tenosynovitis caused by texting: an emerging disease. J Osteop Med 2010;110:294–6. 20538752

[24] Ma T Song L Ning S Relationship between the incidence of de Quervain’s disease among teenagers and mobile gaming. Int Orthop 2019;43:2587–92. 31463625 10.1007/s00264-019-04389-9

[25] Ilyas AM Ast M Schaffer AA De Quervain tenosynovitis of the wrist. J Am Acad Orthop Surg 2007;15:757–64. 18063716 10.5435/00124635-200712000-00009

[26] Gökmen H Gökmen İ Dilek B Addiction of smartphones and related finger deformities: a case report. Turk J Physical Med Rehabil 2020;66:476. 10.5606/tftrd.2020.4256 PMC7756839 33364570

[27] Sharan D Mohandoss M Ranganathan R Musculoskeletal disorders of the upper extremities due to extensive usage of hand held devices. Ann Occup Environ Med 2014;26:22. 25852936 10.1186/s40557-014-0022-3 PMC4387778

[28] Kuss DJ Kanjo E Crook-Rumsey M Problematic mobile phone use and addiction across generations: the roles of psychopathological symptoms and smartphone use. J Technol Behav Sci 2018;3:141–49. 30238057 10.1007/s41347-017-0041-3 PMC6133055

[29] Haug S Castro RP Kwon M Smartphone use and smartphone addiction among young people in Switzerland. J Behav Addict 2015;4:299–307. 26690625 10.1556/2006.4.2015.037 PMC4712764

[30] Gustafsson E Johnson PW Hagberg M. Thumb postures and physical loads during mobile phone use—a comparison of young adults with and without musculoskeletal symptoms. J Electromyogr Kinesiol 2010;20:127–35. 19138862 10.1016/j.jelekin.2008.11.010

[31] Rahaman A Tasnim S Majumdar SH A comprehensive study on excessivein mobile phone use and preventive measures. Int J Mod Educ Comput Sci 2020;12:33-9.

[32] Lee H Kim J. A structural equation model on Korean Adolescents’ excessive use of smartphones. Asian Nurs Res (Korean Soc Nurs Sci) 2018;12:91–8. 10.1016/j.anr.2018.03.002 29614321

[33] Throuvala MA Griffiths MD Rennoldson M Mind over matter: testing the efficacy of an online randomized controlled trial to reduce distraction from smartphone use. Int J Environ Res Public Health 2020;17:4842. 32635650 10.3390/ijerph17134842 PMC7369880

